# Association of blood urea nitrogen to albumin ratio with short-term mortality in critically ill patients with urosepsis

**DOI:** 10.3389/fmed.2026.1860492

**Published:** 2026-08-18

**Authors:** Miao Liu, Xinan Jiang, Chou Yin, Wei Li

**Affiliations:** 1 Department of Urology, Affiliated Hospital of Guizhou Medical University, Guiyang, Guizhou, China; 2 Disease Prevention and Control Center of Yunyan District, Guiyang, China

**Keywords:** 28-day mortality, blood urea nitrogen to albumin ratio, MIMIC-IV database, risk stratification, urosepsis

## Abstract

**Background:**

The incidence of urosepsis is increasing. Early identification of high-risk patients is critical for improving outcomes. The blood urea nitrogen to albumin ratio (BAR) reflects kidney injury and inflammation–nutrition imbalance. However, its prognostic value in patients with urosepsis has not been validated in large-scale studies.

**Methods:**

This study included 3,020 patients with urosepsis from the MIMIC-IV database as the internal discovery cohort, and 415 patients from the Affiliated Hospital of Guizhou Medical University as the external validation cohort. The primary endpoint was 28-day ICU mortality, and the secondary endpoint was 28-day in-hospital mortality. Cox proportional hazards models assessed the association between log-transformed BAR (logBAR) and mortality risk. Restricted cubic splines explored the dose–response relationship. Incremental predictive value was evaluated by adding logBAR to six severity scoring systems (APACHE II, APS III, Charlson, OASIS, SAPS II, SOFA), with DeLong test, NRI, and IDI. The optimal cut-off was determined using the Youden index.

**Results:**

Higher logBAR was independently associated with 28-day ICU mortality (HR = 1.88, 95% CI: 1.58–2.24, *P* < 0.001) and in-hospital mortality (HR = 1.92, 95% CI: 1.60–2.31, *P* < 0.001). A non-linear positive dose–response relationship was observed. Associations were consistent across subgroups (all *P* for interaction > 0.05). Adding logBAR to six scoring systems significantly improved AUC (DeLong *P* < 0.01; ΔAUC 0.024–0.079), with positive NRI (0.289–0.422) and IDI. The optimal cut-off was 2.344 (sensitivity 69.6%, specificity 54.1%). External validation confirmed the association (unadjusted HR = 1.64, *P* = 0.024; adjusted HR = 2.23, 95% CI: 1.29–3.87, *P* = 0.004, EPV = 10.3).

**Conclusion:**

Log-transformed BAR is an independent risk factor for short-term mortality in critically ill patients with urosepsis, showing a non-linear dose–response relationship and good robustness across populations. Adding logBAR to existing severity scoring systems significantly improves predictive performance. This simple, low-cost marker may serve as a useful supplemental tool for risk stratification, although its standalone clinical decision-making utility remains limited. Prospective, multicenter studies are needed to validate these findings.

## Introduction

Sepsis is a complex systemic inflammatory response syndrome triggered by infection. It has a very high mortality rate and has become the leading cause of death in non-cardiac intensive care units (ICUs) (1, 2). This syndrome can affect multiple organs and is influenced by various factors. Although most fatal sepsis cases originate from lower respiratory tract infections, the incidence of sepsis caused by urinary tract infection (UTI) is rising rapidly (3–5). According to reports, about 30% of sepsis cases in the United States are caused by UTI (6). Meanwhile, with the increasing number of urinary system diseases and related surgical procedures, together with the rising antibiotic resistance of pathogens, the disease burden of urosepsis is further increasing (7). In this context, exploring the risk factors for UTI-related bloodstream infection and identifying reliable biomarkers for early recognition of high-risk individuals are crucial for strengthening preventive measures and optimizing treatment strategies.

In recent years, the management of sepsis has seen significant progress, especially in the development of rapid diagnostic tools. These advances have greatly shortened the time needed for pathogen identification, making timely and precise treatment strategies possible (8, 9). Currently, scoring systems such as the Acute Physiology and Chronic Health Evaluation-II (APACHE-II), the Simplified Acute Physiology Score-II (SAPS-II) and the Sequential Organ Failure Assessment (SOFA) are commonly used to assess patient condition. However, these scores have limitations, including cumbersome application, a primary focus on organ physiology and high complexity (10–13). Moreover, these scoring systems were originally designed to predict mortality in general ICU populations, not specifically for patients with urosepsis. Because sepsis from different infection sources has distinct pathophysiological mechanisms (14, 15), there is an urgent clinical need for a simpler, rapid, reproducible and sensitive indicator to assess risk factors and severity of UTI-related bloodstream infection.

Blood urea nitrogen (BUN) and serum albumin (ALB) are routine clinical laboratory markers that are easy to obtain and provide objective results. Previous studies have shown that the BUN to albumin ratio (BAR) reflects kidney function, inflammatory status, nutritional status and endothelial function, and is significantly associated with poor outcomes in various critical illnesses (16–20). However, the role of BAR in predicting outcomes for patients with urosepsis has not been fully studied. Given the unique pathophysiology of urosepsis, BAR, which captures both kidney injury and inflammation-nutrition imbalance, may be particularly relevant (21, 22). Although several studies have reported that BAR is significantly associated with poor prognosis in patients with urosepsis (23), these studies had relatively small sample sizes. Evidence from large-scale populations is still lacking. Therefore, using a public database combined with a real-world cohort for validation, this study aims to systematically evaluate the independent prognostic value of BAR specifically in patients with urosepsis in a large sample population.

## Materials and methods

### Data sources

This study was based on two retrospective cohorts. The internal discovery cohort was derived from the MIMIC-IV database, a publicly available critical care database from the Massachusetts Institute of Technology. This database contains data from 196,527 adult patients admitted to the Beth Israel Deaconess Medical Center between 2008 and 2019 (24). Access to the data was granted after approval of an application. Because this was a retrospective analysis of de-identified information, approval from an institutional review board (IRB) was not required. The external validation cohort included patients with urosepsis admitted to the Department of Urology and the Department of Critical Care Medicine at the Affiliated Hospital of Guizhou Medical University between January 2020 and January 2025. The construction of this cohort was approved by the hospital’s ethics committee.

### Study population

This study included patients admitted to the ICU for sepsis caused by UTI. Sepsis was defined according to the Sepsis-3 consensus criteria and previous literature (25, 26). The diagnostic criteria for UTI differed between cohorts. In the internal discovery cohort, UTI was identified using ICD-9 codes (599.0, 590.10, 590.80, 595.0, 595.9) and ICD-10 codes (N39.0, N10, N11.0, N12, N30.0, N30.9) from the MIMIC-IV database. In the external validation cohort, UTI was diagnosed based on typical clinical manifestations together with positive urine culture results. Patients with UTI-related sepsis were finally included if they met both of the following requirements: a primary diagnosis of UTI, meeting the Sepsis-3 criteria, and the following conditions: (1) age ≥18 years; (2) complete data on serum urea nitrogen and albumin available at admission. It should be noted that the ICD-based definition in the internal cohort and the culture-based definition in the external cohort may not identify fully comparable patient populations. ICD-based definitions may lead to misclassification of infection source, while culture-based definitions may exclude clinically relevant culture-negative cases. This difference is further discussed in the Limitations section. The external validation cohort was initially derived from patients admitted to the Affiliated Hospital of Guizhou Medical University between January 2020 and January 2025. To increase statistical power and ensure robust validation, we extended the recruitment time window to cover from 2015 to January 2026, resulting in a final cohort of 415 patients (compared with the initial 240 patients).

### Data extraction and handling of missing data

After obtaining the necessary permissions, data were extracted using PostgreSQL (13.7.2), Navicat Premium (16.0) and structured query language (SQL). The extracted variables were classified into six categories: demographic characteristics, comorbidities, vital signs, laboratory parameters, disease severity scores and treatment strategies. Detailed definitions of each variable are provided in Supplementary Table 1. The exposure variable of interest was the BAR, calculated as BAR = BUN (mg/dL)/ALB (g/dL). Because BAR showed a markedly skewed distribution, it was natural log-transformed (logBAR) for all subsequent analyses. The primary endpoint was 28-day ICU mortality, and the secondary endpoint was 28-day in-hospital all-cause mortality. For missing data, variables with more than 30% missing values were first excluded. For the remaining variables with missing values, multiple imputation was performed using the “mice” package (version 3.16.0) in R. The imputation model included all variables from the main analysis: continuous variables requiring imputation, the complete exposure variable (BAR and its components), the complete outcome variable (28-day mortality), and other complete categorical and continuous covariates. This strategy used available information to predict missing values, strengthening the plausibility of the missing at random (MAR) assumption. In addition, to improve transparency, the missing rates for each analytical variable and the core code for imputation are provided in the Supplementary file.

### Association between logBAR and endpoints

After testing the proportional hazards assumption, Cox proportional hazards models were used to analyse the association between logBAR and clinical endpoints. Three progressively adjusted models were constructed to control for confounders. Model 1 included logBAR only (unadjusted). Model 2 additionally adjusted for demographic characteristics (age, sex, race, weight) and baseline comorbidities that differed between groups, including acute kidney injury (AKI), chronic kidney disease (CKD), hyperlipidaemia (HLD), heart failure (HF), myocardial infarction (MI) and chronic obstructive pulmonary disease (COPD). Model 3 further adjusted for additional key laboratory parameters and treatments based on all variables in Model 2, after excluding multicollinearity (variance inflation factor > 5; all VIF values are shown in Table 1) and with reference to previous literature (24–28). These additional variables included: systolic blood pressure (NBPS), diastolic blood pressure (NBPD), oxygen saturation (Spo2), red blood cell count (RBC), white blood cell count (WBC), anion gap (GA), serum potassium (K), total carbon dioxide (CO2CP), blood lactate (Lac), pH, partial pressure of oxygen (PO2), partial thromboplastin time (PTT), alanine aminotransferase (ALT), total bilirubin (TB), serum creatinine (CREA), lactate dehydrogenase (LDH), analgesia/sedation (SA, a binary treatment variable, not serum albumin), use of vasopressors (VP), use of glucocorticoids (GC) and continuous renal replacement therapy (CRRT). Subsequently, restricted cubic splines (RCS, with knots at the 5th, 35th, 65th and 95th percentiles) were used to explore the non-linear relationship between logBAR and endpoints. In addition, Kaplan-Meier survival curves were used to further validate the association between logBAR and poor prognosis in patients with urosepsis.

**TABLE 1:** Baseline characteristics of critically ill patients with urosepsis stratified by tertiles of log-transformed blood urea nitrogen to albumin ratio (logBAR) in the MIMIC-IV cohort.

	ALL	Low	Moderate	High	*P*-value
Variables	*N = 3020*	*N = 1007*	*N = 1007*	*N = 1006*	
Age	70.5 (15.1)	67.5 (16.2)	72.0 (14.2)	72.0 (14.4)	<0.001
Gender:	1318 (43.6%)	382 (37.9%)	443 (44.0%)	493 (49.0%)	<0.001
Race:	1962 (65.0%)	642 (63.8%)	663 (65.8%)	657 (65.3%)	0.595
Weight	82.1 (25.8)	80.1 (23.5)	82.6 (26.8)	83.7 (26.9)	0.003
Hpy:	1062 (35.2%)	470 (46.7%)	370 (36.7%)	222 (22.1%)	<0.001
AKI:	1896 (62.8%)	351 (34.9%)	691 (68.6%)	854 (84.9%)	<0.001
CKD:	878 (29.1%)	111 (11.0%)	293 (29.1%)	474 (47.1%)	<0.001
DM:	1141 (37.8%)	288 (28.6%)	403 (40.0%)	450 (44.7%)	<0.001
HLD:	1059 (35.1%)	335 (33.3%)	380 (37.7%)	344 (34.2%)	0.085
HF:	1189 (39.4%)	279 (27.7%)	439 (43.6%)	471 (46.8%)	<0.001
MI:	317 (10.5%)	75 (7.45%)	104 (10.3%)	138 (13.7%)	<0.001
IHD:	1115 (36.9%)	302 (30.0%)	383 (38.0%)	430 (42.7%)	<0.001
COPD:	495 (16.4%)	146 (14.5%)	190 (18.9%)	159 (15.8%)	0.025
SOFA	6.90 (3.48)	5.69 (2.98)	6.94 (3.38)	8.08 (3.62)	<0.001
APSIII	58.9 (21.0)	48.3 (17.8)	59.3 (18.8)	69.2 (20.8)	<0.001
SAPSII	45.0 (13.4)	38.7 (11.8)	45.8 (12.2)	50.5 (13.3)	<0.001
OASIS	36.0 (8.32)	34.8 (7.71)	36.3 (8.31)	36.8 (8.79)	<0.001
Charlson	6.31 (2.91)	5.19 (2.81)	6.57 (2.74)	7.16 (2.83)	<0.001
APACHEII	21.8 (7.00)	18.7 (6.23)	21.6 (6.53)	25.0 (6.77)	<0.001
HR	91.5 (21.3)	90.9 (21.4)	91.7 (20.7)	91.8 (21.8)	0.604
NBPS	120 (25.8)	125 (26.6)	117 (25.4)	117 (24.6)	<0.001
NBPD	67.5 (19.8)	70.4 (19.1)	66.1 (19.0)	66.0 (20.8)	<0.001
RR	20.2 (6.41)	19.6 (6.11)	20.3 (6.39)	20.7 (6.68)	0.001
Spo2	96.5 (4.73)	96.6 (4.69)	96.4 (4.65)	96.4 (4.86)	0.447
HCT	31.3 (6.63)	33.0 (6.43)	31.3 (6.70)	29.6 (6.32)	<0.001
Hb	10.1 (2.20)	10.8 (2.16)	10.1 (2.20)	9.48 (2.05)	<0.001
PLT	200 (118)	207 (117)	199 (116)	195 (120)	0.069
RDW	16.1 (2.63)	15.4 (2.51)	16.1 (2.55)	16.8 (2.61)	<0.001
RBC	3.40 (0.78)	3.62 (0.77)	3.38 (0.76)	3.19 (0.73)	<0.001
WBC	13.9 (14.8)	12.7 (12.6)	13.9 (11.9)	15.0 (19.0)	0.005
ALB	2.90 (0.62)	3.13 (0.59)	2.90 (0.58)	2.68 (0.60)	<0.001
GA	15.7 (4.73)	14.3 (3.90)	15.2 (4.49)	17.6 (5.12)	<0.001
Ca	8.27 (0.99)	8.32 (0.96)	8.27 (0.93)	8.23 (1.07)	0.139
Cl	104 (7.94)	104 (6.98)	104 (7.28)	104 (9.35)	0.739
Glu	154 (84.9)	145 (65.7)	157 (89.1)	159 (96.2)	<0.001
K	4.23 (0.80)	3.96 (0.67)	4.24 (0.78)	4.48 (0.85)	<0.001
Na	139 (6.77)	138 (5.67)	138 (6.08)	139 (8.27)	0.381
CO2CP	23.9 (6.52)	25.1 (5.91)	24.2 (6.51)	22.5 (6.84)	<0.001
LAC	2.43 (2.04)	2.23 (1.76)	2.52 (2.12)	2.54 (2.21)	<0.001
PCO2	42.3 (12.7)	42.5 (12.9)	43.0 (12.7)	41.3 (12.4)	0.007
PH	7.35 (0.10)	7.37 (0.10)	7.35 (0.10)	7.33 (0.11)	<0.001
PO2	117 (96.5)	139 (109)	113 (94.3)	98.3 (80.1)	<0.001
INR	1.40 [1.20; 1.80]	1.30 [1.10; 1.60]	1.40 [1.20; 1.80]	1.50 [1.20; 1.90]	<0.001
PT	15.1 [13.0; 19.1]	14.1 [12.6; 17.1]	15.4 [13.1; 19.6]	15.9 [13.5; 20.9]	<0.001
PTT	32.4 [27.9; 41.4]	31.3 [27.2; 39.5]	32.7 [28.5; 41.6]	33.5 [28.1; 43.0]	<0.001
ALT	25.0 [15.0; 54.0]	24.0 [15.0; 50.0]	26.0 [15.0; 56.0]	27.0 [15.0; 58.0]	0.091
AST	40.0 [23.0; 87.0]	37.0 [23.0; 73.5]	42.0 [24.0; 91.5]	42.0 [23.2; 95.0]	0.025
TB	0.70 [0.40; 1.40]	0.60 [0.40; 1.20]	0.70 [0.40; 1.50]	0.70 [0.40; 1.70]	0.042
CREA	1.30 [0.80; 2.20]	0.80 [0.60; 1.10]	1.40 [1.00; 1.80]	2.50 [1.70; 3.90]	<0.001
UREA	29.0 [18.0; 49.0]	15.0 [11.0; 18.0]	29.0 [24.0; 35.0]	60.5 [47.0; 82.0]	<0.001
LDH	285 [213; 415]	279 [214; 400]	285 [210; 409]	298 [214; 439]	0.041
SA	2032 (67.3%)	713 (70.8%)	678 (67.3%)	641 (63.7%)	0.003
VP:	1880 (62.3%)	557 (55.3%)	656 (65.1%)	667 (66.3%)	<0.001
GC:	980 (32.5%)	291 (28.9%)	331 (32.9%)	358 (35.6%)	0.006
Ventilation:	2610 (86.4%)	893 (88.7%)	876 (87.0%)	841 (83.6%)	0.003
CRRT:	326 (10.8%)	42 (4.17%)	105 (10.4%)	179 (17.8%)	<0.001
Hosp dead	406 (13.4%)	56 (5.56%)	138 (13.7%)	212 (21.1%)	<0.001
ICU dead	444 (14.7%)	63 (6.26%)	155 (15.4%)	226 (22.5%)	<0.001

Data are presented as mean (SD), median [IQR], or *n* (%). *P*-values were calculated using ANOVA, Kruskal-Wallis test, chi-square test, or Fisher’s exact test as appropriate. AKI, acute kidney injury; CKD, chronic kidney disease; DM, diabetes mellitus; HLD, hyperlipidemia; HF, heart failure; MI, myocardial infarction; IHD, ischemic heart disease; COPD, chronic obstructive pulmonary disease; SOFA, Sequential Organ Failure Assessment; APS III, Acute Physiology Score III; SAPS II, Simplified Acute Physiology Score II; OASIS, Oxford Acute Severity of Illness Score; APACHE II, Acute Physiology and Chronic Health Evaluation II; HR, heart rate; NBPS, non-invasive blood pressure systolic; NBPD, non-invasive blood pressure diastolic; RR, respiratory rate; Spo2, oxygen saturation; HCT, hematocrit; Hb, hemoglobin; PLT, platelet count; RDW, red cell distribution width; RBC, red blood cell count; WBC, white blood cell count; ALB, albumin; GA, anion gap; Ca, calcium; Lac, lactate; PCO2, partial pressure of carbon dioxide; PH, arterial pH; PO2, partial pressure of oxygen; INR, international normalized ratio; PT, prothrombin time; PTT, partial thromboplastin time; ALT, alanine aminotransferase; AST, aspartate aminotransferase; TB, total bilirubin; CREA, creatinine; UREA, urea; LDH, lactate dehydrogenase; SA, analgesia/sedation; VP, vasopressors; GC, glucocorticoids; CRRT, continuous renal replacement therapy; Hosp dead, in-hospital death; ICU dead, intensive care unit death.

### Incremental effect of logBAR

Log-transformed BAR was added to each of six commonly used critical care scoring systems, and multivariable logistic regression models were constructed. Based on the regression coefficients of each variable, a composite risk score was calculated using the formula (β_1_ × variable1 + β_2_ × variable_2_). Receiver operating characteristic (ROC) curves were plotted and the area under the curve (AUC) was calculated to evaluate the predictive ability of each model for poor outcomes. The DeLong test was used to compare the predictive performance of models with and without logBAR, to determine whether the improvement was statistically significant.

### Subgroup and interaction analyses of logBAR

To test the robustness of the association between logBAR and clinical endpoints, pre-specified subgroup analyses and interaction tests were performed. Subgroups were defined based on key characteristics including age, sex, race and baseline comorbidities. The statistical significance of interaction terms (P for interaction) was calculated to assess whether the effect of logBAR was consistent across subgroups. If none of the interaction terms was significant (generally *P* > 0.05), this indicated good stability of the association across different populations. Conversely, a significant interaction suggested heterogeneity. All analyses followed a pre-specified protocol to control for bias from multiple comparisons.

### Calibration and decision curve analysis

To assess model calibration, we constructed calibration plots by comparing observed 28-day ICU mortality rates against the predicted probabilities from each model (i.e., severity score alone versus score plus logBAR). Calibration slope and intercept were derived from a logistic regression of the actual outcome on the logit of the predicted risk, where a perfectly calibrated model would yield a slope of 1 and an intercept of 0. For both the internal and external validation cohorts, we estimated optimism-corrected calibration slopes and intercepts via 500 bootstrap resamples, fitting the models separately within each cohort following the same stepwise incremental approach used in the AUC analyses. Additionally, we tabulated observed versus predicted mortality across deciles of predicted risk. To gauge clinical usefulness, decision curve analysis (DCA) was employed, quantifying the net benefit across threshold probabilities ranging from 0.01 to 0.50. At each threshold, the net benefit of the score-alone model was compared with that of the score-plus-logBAR model, using treat-all and treat-none as reference strategies. DCA was applied to both cohorts. All calibration analyses were carried out with the rms package, and DCA with the dcurves package in R.

### Statistical analysis

Log-transformed BAR in the baseline table was divided into three groups (low, medium and high) according to tertiles. Continuous variables were described as mean ± standard deviation or median (interquartile range) depending on their distribution, and comparisons between groups were performed using Student’s *t*-test, ANOVA or the Kruskal-Wallis test as appropriate. Categorical variables were presented as frequencies (percentages), and differences between groups were tested using the Pearson chi-square test or Fisher’s exact test. All statistical analyses were performed using R software (version 4.5.1). A two-sided *P*-value < 0.05 was considered statistically significant.

## Results

### Baseline patient characteristics

According to the strict inclusion criteria, a total of 3,020 critically ill patients with urosepsis were included in this study. The mean age was 70.5 years, and 43.6% (1,318 patients) were male. After stratifying patients by logBAR tertiles, baseline characteristics are shown in the table. Compared with the low logBAR group, patients in the high logBAR group were older (72.0 years vs. 67.5 years) and had higher proportions of AKI, CKD, DM, HF and ischaemic cardiomyopathy. Regarding treatments, the proportions of patients receiving VP, GC and CRRT were significantly higher in the high logBAR group. In terms of vital signs, the high logBAR group showed lower NBPD and NBPS and a higher respiratory rate. Laboratory results indicated that the high logBAR group had elevated levels of RDW, RBC, K, Lac, INR, PTT, PT, CRE and URE. In contrast, levels of Hb, RBC, ALB, CO2CP, pH and PO2 were decreased. All clinical severity scores, including SOFA, APS III, SAPS II, Charlson comorbidity index and APACHE II, were positively correlated with logBAR levels. Regarding prognosis, mortality was significantly higher in the high logBAR group than in the low logBAR group: ICU mortality was 22.5% vs. 6.26% (*P* < 0.001), and in-hospital mortality was 21.1% vs. 5.56% (*P* < 0.001).

### Association between logBAR and short-term mortality in critically ill patients with urosepsis

As shown in Table 2, higher logBAR was significantly associated with 28-day ICU mortality in all three Cox regression models: unadjusted Model 1 (HR = 2.14, 95% CI: 1.89–2.42, *P* < 0.001), Model 2 adjusting for demographics and comorbidities (HR = 1.85, 95% CI: 1.60–2.13, *P* < 0.001), and Model 3 with further adjustment for clinical variables (HR = 1.88, 95% CI: 1.58–2.24, *P* < 0.001). Using the low logBAR group as the reference, the medium and high logBAR groups showed significantly increased mortality risk in all models. In Model 1, the HR for the medium group was 2.88 (2.15–3.86) and for the high group was 4.58 (3.46–6.06), both *P* < 0.001. In Model 2, the HR for the medium group was 2.30 (1.69–3.12) and for the high group was 3.32 (2.44–4.51), both *P* < 0.001. In Model 3, the HR for the medium group was 2.07 (1.52–2.83) and for the high group was 2.94 (2.11–4.10), both *P* < 0.001.

**TABLE 2:** Cox proportional hazards regression analysis for the association between logBAR and 28-day ICU mortality in the MIMIC-IV cohort.

	Model 1			Model 2			Model 3		
Characteristic	HR	95% CI	*P*-value	HR	95% CI	*P*-value	HR	95% CI	*P*-value
logBAR	2.14	1.89–2.42	<0.001	1.85	1.60–2.13	<0.001	1.88	1.58–2.24	<0.001
logBAR group									
Low	Ref	Ref		Ref	Ref		Ref	Ref	
Moderate	2.88	2.15–3.86	<0.001	2.30	1.69–3.12	<0.001	2.07	1.52–2.83	<0.001
High	4.58	3.46–6.06	<0.001	3.32	2.44–4.51	<0.001	2.94	2.11–4.10	<0.001

CI, confidence interval; HR, hazard ratio. Model 1: unadjusted. Model 2: adjusted for age, sex, race, weight, AKI, CKD, HLD, HF, MI, COPD. Model 3: further adjusted for NBPS, NBPD, Spo2, RBC, WBC, GA, K, TCO2, Lac, PH, PO2, PTT, ALT, TB, CREA, LDH, SA (analgesia/sedation), VP, GC, CRRT.

**FIGURE 1:**
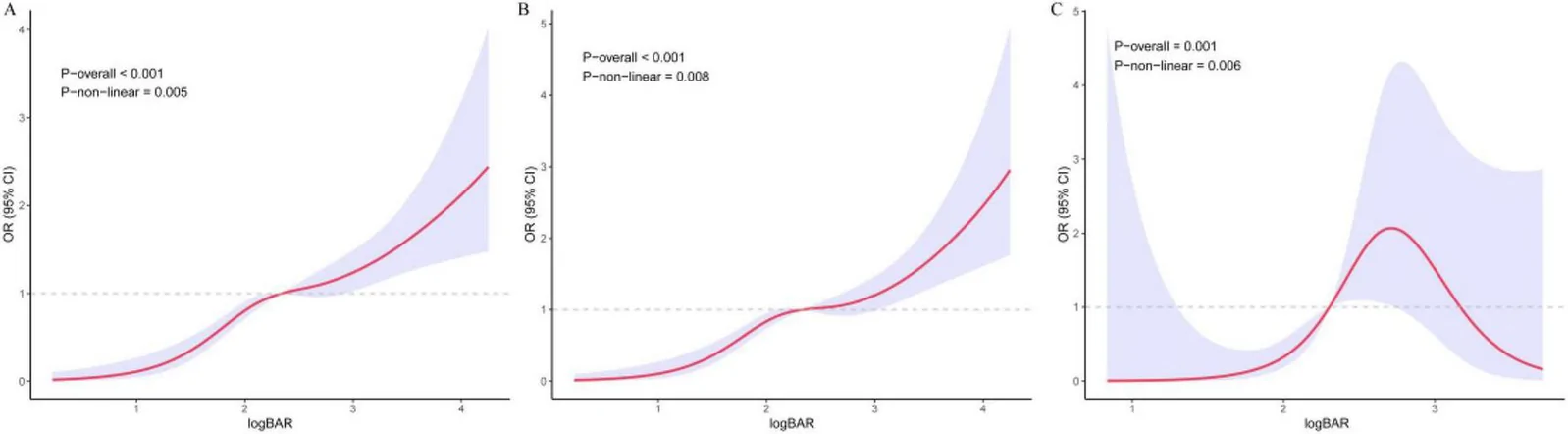
Dose–response relationship between logBAR and short-term mortality in patients with urosepsis, estimated by restricted cubic splines with knots at the 5th, 35th, 65th and 95th percentiles. **(A)** 28-day ICU mortality in the MIMIC-IV cohort (*n* = 3,020). **(B)** 28-day in-hospital mortality in the MIMIC-IV cohort. **(C)** 28-day ICU mortality in the external validation cohort (*n* = 415). Solid lines represent the hazard ratio (HR) relative to the reference logBAR value (median), and shaded areas indicate the 95% confidence interval. The distribution of logBAR is shown as tick marks along the *x*-axis.

**FIGURE 2:**
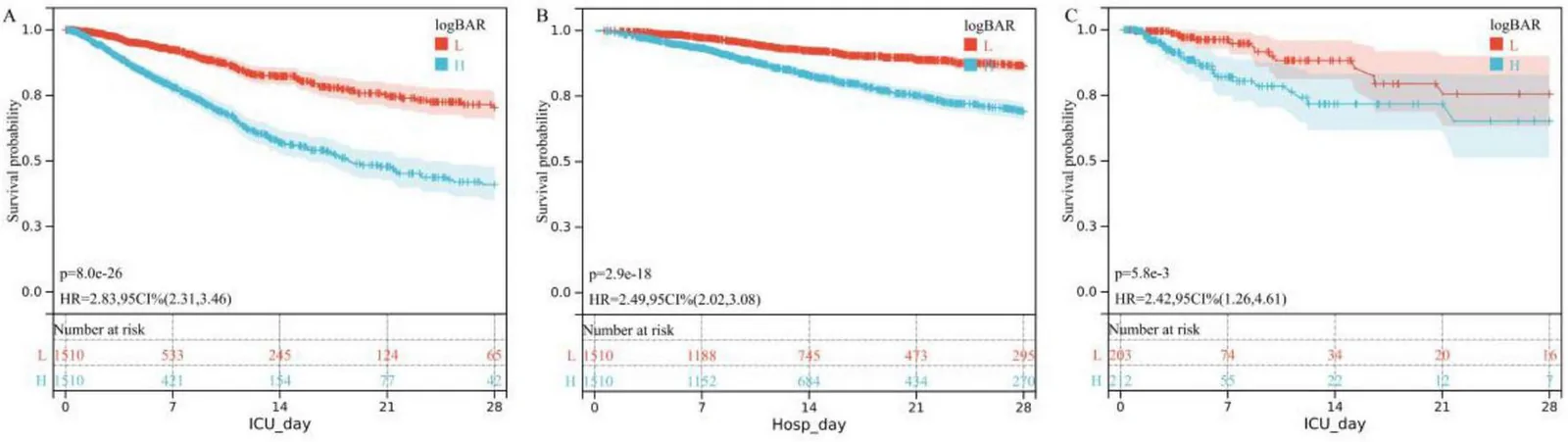
Kaplan-Meier survival curves for short-term mortality according to logBAR tertiles. **(A)** ICU survival probability over 28 days in the MIMIC-IV cohort. **(B)** In-hospital survival probability over 28 days in the MIMIC-IV cohort. **(C)** ICU survival probability over 28 days in the external validation cohort. The log-rank test was used to compare differences among groups. Number of patients at risk at each time point is shown below ach panel.

Similarly, as shown in Table 3, for in-hospital mortality, logBAR as a continuous variable indicated increased risk in all models: Model 1 HR = 2.05 (1.80–2.34), Model 2 HR = 1.80 (1.55–2.10), Model 3 HR = 1.92 (1.60–2.31), all *P* < 0.001. In the categorical analysis, compared with the low logBAR group, the medium group had HRs of 2.66 (1.95–3.63), 2.14 (1.55–2.96) and 2.01 (1.45–2.79) in Models 1, 2 and 3, respectively, all *P* < 0.001. The high group had HRs of 4.13 (3.08–5.55), 3.07 (2.23–4.25) and 2.99 (2.11–4.26), all *P* < 0.001. The RCS curves further showed a non-linear positive dose–response relationship between logBAR and short-term mortality in critically ill patients with urosepsis (Figures 1A, B). Further KM survival curves indicated that higher logBAR was associated with shorter survival times (Figures 2A,B).

**TABLE 3:** Cox proportional hazards regression analysis for the association between logBAR and 28-day in-hospital all-cause mortality in the MIMIC-IV cohort.

	Model 1			Model 2			Model 3		
Characteristic	HR	95% CI	*P*-value	HR	95% CI	*P*-value	HR	95% CI	*P*-value
logBAR	2.05	1.80–2.34	<0.001	1.83	1.58–2.13	<0.001	1.93	1.60–2.32	<0.001
logBAR group									
Low	Ref	Ref		Ref	Ref		Ref	Ref	
Moderate	2.66	1.95–3.63	<0.001	2.17	1.58–3.00	<0.001	2.02	1.46–2.81	<0.001
High	4.13	3.08–5.55	<0.001	3.17	2.30–4.37	<0.001	3.02	2.12–4.29	<0.001

CI, confidence interval; HR, hazard ratio. Model 1: unadjusted. Model 2: adjusted for age, sex, race, weight, AKI, CKD, HLD, HF, MI, COPD. Model 3: further adjusted for NBPS, NBPD, Spo2, RBC, WBC, GA, K, TCO2, Lac, PH, PO2, PTT, ALT, TB, CREA, LDH, SA (analgesia/sedation), VP, GC, CRRT.

### Incremental effect of logBAR

**FIGURE 3:**
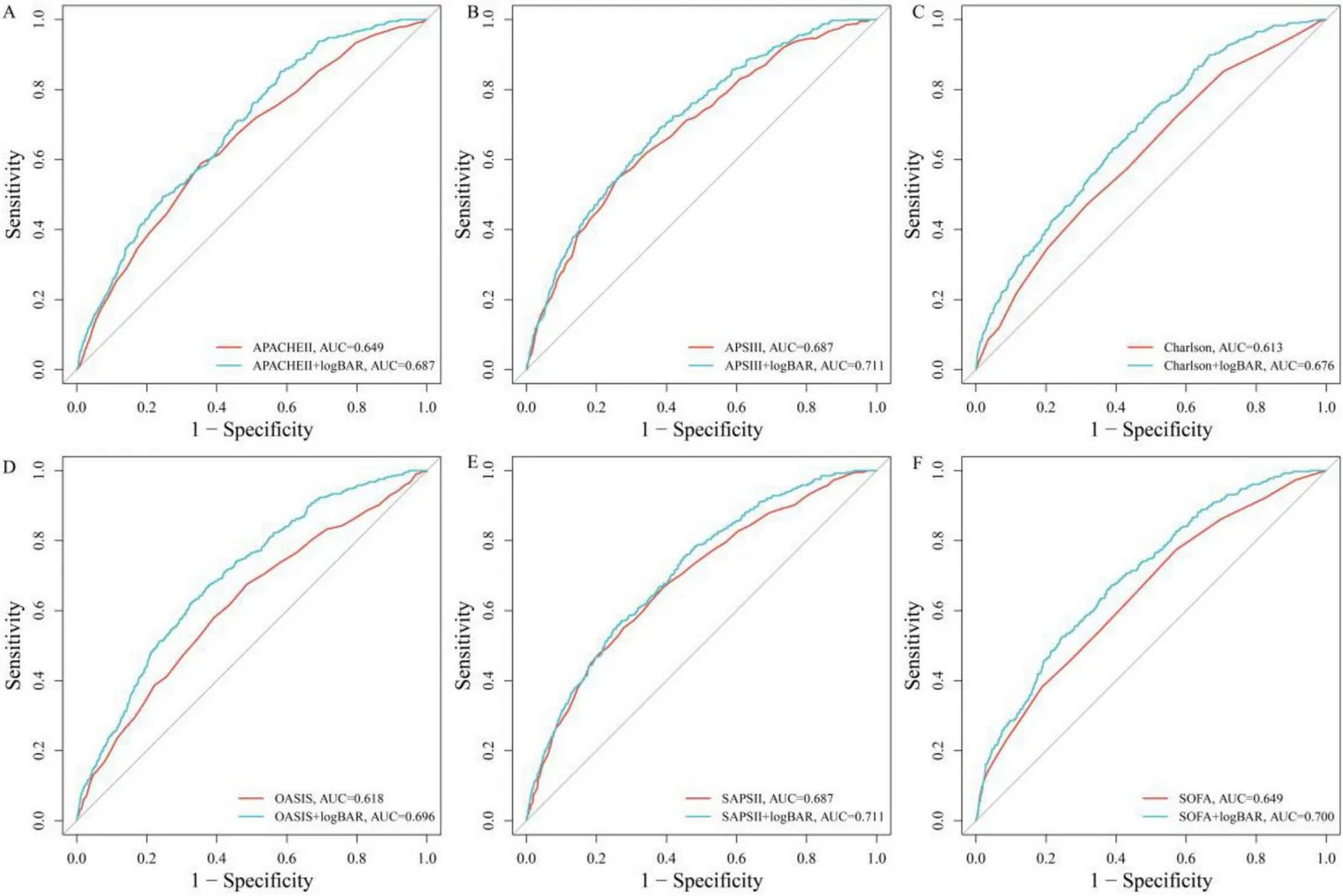
Incremental predictive value of logBAR added to six traditional severity scoring systems for 28-day ICU mortality. Receiver operating characteristic (ROC) curves for models containing each severity score alone (dashed lines) and the same score plus logBAR (solid lines). (A) APACHE II; (B) APS III; (C) Charlson comorbidity index; (D) OASIS; (E) SAPS II; (F) SOFA. The area under the curve (AUC) is shown for each model. The DeLong test was used to compare AUCs between the score-alone model and the score-plus-logBAR model (all *P* < 0.01). The diagonal gray line represents the reference of no discrimination (AUC = 0.5).

We used AUC analysis to evaluate the impact of adding logBAR to existing disease severity scores (APACHE II, APS III, Charlson, OASIS, SAPS II and SOFA) for predicting 28-day ICU mortality. As shown in Table 4 and Figures 3A–F, for ICU mortality, adding logBAR consistently improved the predictive performance of all scores (all DeLong test *P* < 0.01): APACHE II increased from 0.648 to 0.685 (Figure 3A), APS III from 0.691 to 0.712 (Figure 3B), Charlson comorbidity index from 0.614 to 0.675 (Figure 3C), OASIS from 0.609 to 0.688 (Figure 3D), SAPS II from 0.688 to 0.712 (Figure 3E) and SOFA from 0.647 to 0.697 (Figure 3F).

**TABLE 4:** Incremental predictive value of logBAR added to six conventional severity scoring systems for predicting 28-day mortality in the MIMIC-IV cohort.

Variable	Δ AUC	DeLong P	NRI (95% CI)	IDI (95% CI)	Δ Brier
28-day ICU mortality					
APACHE II	0.037	<0.001	0.338 (0.239, 0.443)	0.0220 (0.0157, 0.0289)	−0.0026
APS III	0.021	0.002	0.339 (0.233, 0.440)	0.0148 (0.0092, 0.0208)	−0.0018
Charlson	0.061	<0.001	0.422 (0.335, 0.518)	0.0310 (0.0244, 0.0387)	−0.0038
OASIS	0.079	<0.001	0.384 (0.289, 0.480)	0.0383 (0.0304, 0.0468)	−0.0045
SAPS II	0.024	0.001	0.289 (0.190, 0.396)	0.0160 (0.0103, 0.0223)	−0.0019
SOFA	0.050	<0.001	0.375 (0.279, 0.469)	0.0241 (0.0172, 0.0318)	−0.0028
28-day in-hospital mortality					
APACHE II	0.038	<0.001	0.353 (0.247, 0.460)	0.0232 (0.0163, 0.0305)	−0.0026
APS III	0.024	<0.001	0.343 (0.234, 0.446)	0.0160 (0.0098, 0.0224)	−0.0019
Charlson	0.063	<0.001	0.419 (0.320, 0.516)	0.0323 (0.0256, 0.0402)	−0.0038
OASIS	0.078	<0.001	0.392 (0.290, 0.496)	0.0380 (0.0299, 0.0468)	−0.0041
SAPS II	0.024	0.002	0.297 (0.189, 0.407)	0.0175 (0.0117, 0.0238)	−0.0020
SOFA	0.051	<0.001	0.396 (0.295, 0.499)	0.0248 (0.0175, 0.0329)	−0.0027

ΔAUC, difference in AUC; DeLong P, *P*-value from DeLong test; NRI, net reclassification improvement (category-free, bootstrap 95% CI); IDI, integrated discrimination improvement (bootstrap 95% CI); ΔBrier, difference in Brier score (negative indicates improvement). All DeLong tests and NRI/IDI were significant (*P* < 0.05).

**FIGURE 4:**
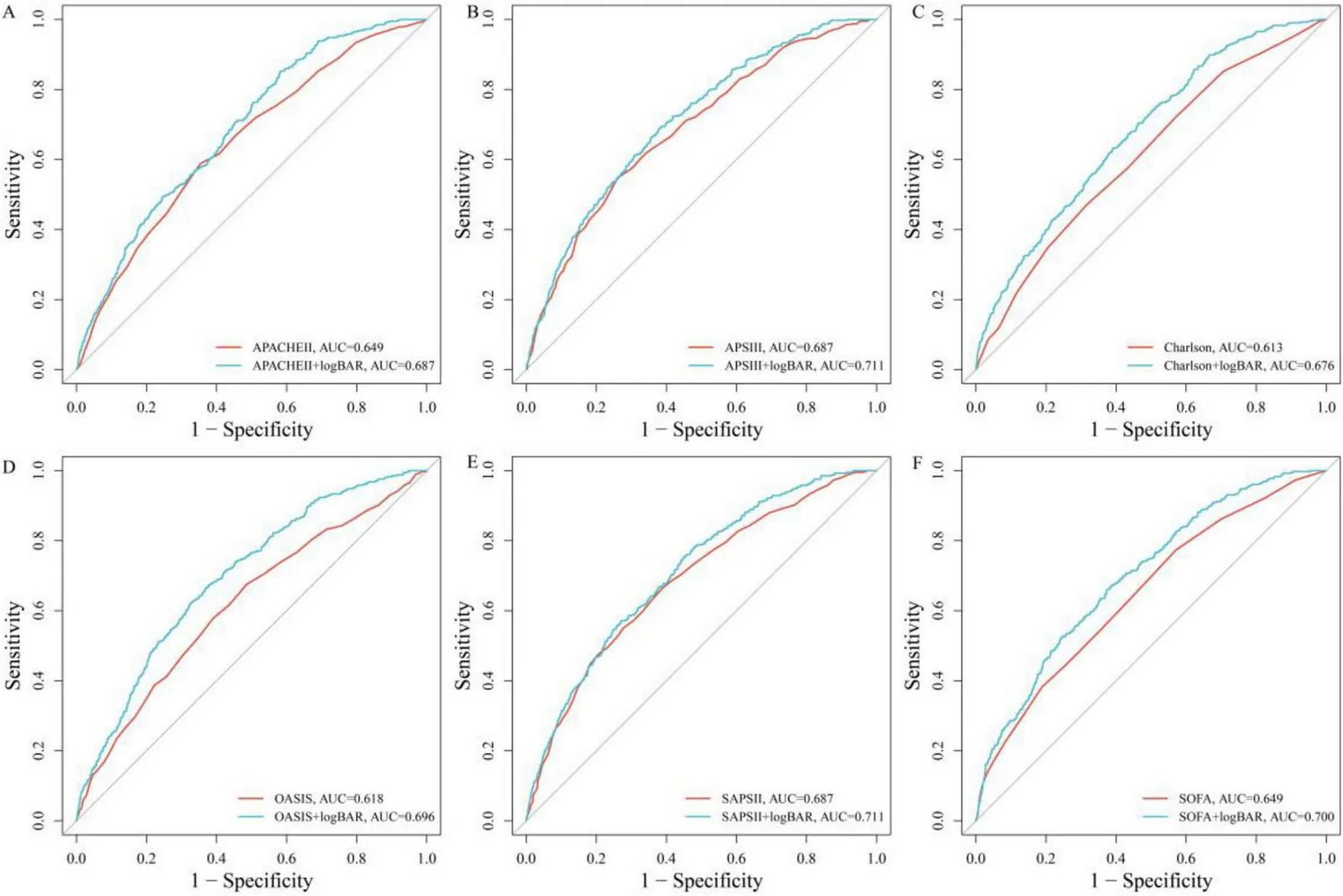
Incremental predictive value of logBAR added to six traditional severity scoring systems for 28-day in-hospital all-cause mortality. ROC curves for models with each severity score alone (dashed lines) and with the addition of logBAR (solid lines). (A) APACHE II; (B) APS III; (C) Charlson comorbidity index; (D) OASIS; (E) SAPS II; (F) SOFA. AUC values are provided for each model. All comparisons by DeLong test showed significant improvement after adding logBAR (*P* < 0.01). The diagonal reference line indicates AUC = 0.5.

Similarly, for in-hospital mortality, adding logBAR consistently improved the predictive performance of all scores (Table 4; all DeLong test *P* < 0.01): APACHE II increased from 0.649 to 0.687 (Figure 4A), APS III from 0.687 to 0.711 (Figure 4B), Charlson comorbidity index from 0.613 to 0.676 (Figure 4C), OASIS from 0.618 to 0.696 (Figure 4D), SAPS II from 0.687 to 0.711 (Figure 4E) and SOFA from 0.649 to 0.700 (Figure 4F). These findings were further supported by net reclassification improvement (NRI) and integrated discrimination improvement (IDI) analyses (Table 4): all NRI values were positive (ranging from 0.289 to 0.422 for ICU mortality and from 0.297 to 0.419 for in-hospital mortality), and all IDI values were positive (ranging from 0.0148 to 0.0383 for ICU mortality), confirming that adding logBAR significantly improved reclassification and discrimination beyond AUC improvement alone. Brier scores were also consistently reduced (ΔBrier ranging from -0.0045 to -0.0018), indicating improved overall prediction accuracy. These findings further highlight the potential clinical value of logBAR. Calibration was well maintained internally (slopes near 1, intercepts near 0), with logBAR not impairing calibration; external validation (*n* = 41) showed variable slopes but improved toward 1.0 after adding logBAR. Decision curve analysis revealed modest net benefit internally at thresholds 5%–30%, yet negligible externally, suggesting that logBAR’s independent clinical utility may be limited in external populations. These findings further highlight the potential clinical value of logBAR (Supplementary Figures 3, 4).

### Subgroup and interaction analyses

To explore whether demographic characteristics and comorbidities influenced the association between logBAR and 28-day ICU/in-hospital mortality, subgroup analyses and interaction tests were performed. After multivariable adjustment (Supplementary Figures 1, 2), the association between logBAR and short-term mortality in patients with urosepsis remained consistent across subgroups of different demographic characteristics (age, sex, race) and comorbidities (hypertension, AKI, DM, CKD, HLD, HF, MI, IHD, COPD). Interaction tests showed no significant interaction between logBAR and any of these stratification factors (all *P* for interaction > 0.05), indicating that the predictive effect of logBAR on mortality risk was stable and consistent across different subgroups.

### Clinical value interpretation of logBAR

To enhance the clinical utility of logBAR, we determined the optimal cut-off value of logBAR for predicting 28-day ICU mortality in the internal MIMIC-IV cohort (*n* = 3,020) using the ROC curve and maximizing the Youden index. The optimal cut-off value was 2.344. This cut-off corresponded to a sensitivity of 69.6%, a specificity of 54.1% and a Youden index of 0.237. This means that using this cut-off could identify about 69.6% of patients at risk of death while correctly excluding about 54.1% of survivors. Although this marker has moderate sensitivity, its low specificity suggests that using logBAR alone as a precise diagnostic or decision-making tool has limitations, with a relatively high false positive rate.

### External cohort validation

As shown in Supplementary Table 2, the external validation cohort included 415 patients with urosepsis (28-day mortality rate = 9.88%). The logBAR level was significantly higher in non-survivors than in survivors (2.50 ± 0.40 vs. 2.34 ± 0.75, *P* = 0.026). In unadjusted Cox regression (Model 1), higher logBAR as a continuous variable was significantly associated with increased 28-day mortality risk (HR = 1.64, 95% CI: 1.07–2.51, *P* = 0.024). Because the external cohort had only 41 events, the full Model 3 with 29 covariates yielded an events-per-variable (EPV) ratio of 1.41, far below the recommended threshold of 10, indicating a high risk of overfitting. To obtain a statistically stable estimate, we additionally fitted a reduced multivariable model adjusted for age, sex, lactate, and serum creatinine (4 covariates; EPV = 10.3). In this reduced model, higher logBAR remained significantly associated with increased 28-day mortality risk (HR = 2.23, 95% CI: 1.29–3.87, *P* = 0.004). The full Model 3 result (HR = 2.90, 95% CI: 1.40–6.01, *P* = 0.004) is reported as a sensitivity analysis but should be interpreted with caution given the inadequate EPV. When the optimal cut-off of logBAR = 2.344 derived from the internal cohort was applied to the external cohort, the sensitivity was 65.9%, specificity was 52.9%, PPV was 13.3%, and NPV was 93.4%. The high NPV but low PPV suggests that this cut-off may be useful for ruling out low-risk patients but is not suitable as a standalone tool for confirming high-risk status. The cut-off should be considered exploratory pending prospective validation in larger multicenter cohorts. The RCS curve confirmed a significant non-linear positive dose–response relationship between logBAR and short-term ICU mortality (*P* < 0.001; Figure 1C). KM survival curves further confirmed that patients in the high logBAR group had shorter survival times (Figure 2C).

## Discussion

Urosepsis is a serious complication of urinary tract infection. Its incidence has been increasing in recent years, making it a major cause of the growing sepsis population and placing a heavy burden on individual patients and public health systems (5–7). Although the management of sepsis has seen significant progress in recent years, particularly in the development of rapid diagnostic tools, advances that have greatly shortened the time needed for pathogen identification and enabled timely, precise treatment strategies (8, 9), various critical care indicators commonly used in clinical practice have limitations. These include cumbersome application, a primary focus on organ physiology and high complexity, which may still cause high-risk patients to miss the optimal window for intervention (10–13). Therefore, there is an urgent clinical need for a simpler, rapid, reproducible and sensitive indicator to assess risk factors and severity of UTI-related bloodstream infection.

Blood urea nitrogen and ALB are both routine clinical laboratory markers. Elevated BUN not only reflects reduced glomerular filtration function but also sensitively indicates pre-renal factors and metabolic load under the hypercatabolic state of sepsis (29). Albumin, as a comprehensive surrogate marker of nutritional and inflammatory status, decreases because of reduced synthesis and capillary leakage (30). Using either marker alone cannot fully assess disease severity. In contrast, BAR combines both and systematically captures the dual pathological axis of kidney injury and inflammation-nutrition imbalance, which is theoretically more suitable for prognostic assessment in urosepsis. The reason is that urosepsis is driven mainly by Gram-negative bacterial endotoxins and obstruction-reflux, with more prominent tubular injury and systemic inflammatory response; the incidence of AKI is higher than in pulmonary or blood-borne sepsis (31, 32). BAR exactly reflects both tubular injury (BUN) and inflammation-nutrition status (albumin), providing unique pathophysiological relevance.

To test this hypothesis, the present study used two independent large retrospective cohorts (the internal discovery cohort from MIMIC-IV and the external validation cohort from the Affiliated Hospital of Guizhou Medical University). For the first time in a relatively large sample of critically ill patients with urosepsis, we demonstrated that elevated logBAR was an independent risk factor for 28-day ICU mortality and 28-day in-hospital all-cause mortality (HR = 1.88, 95% CI: 1.58–2.24, *P* < 0.001). Restricted cubic spline curves showed a non-linear positive dose–response relationship between logBAR and short-term mortality. Kaplan–Meier survival curves further indicated that patients in the high logBAR group had significantly shorter survival times. These associations were consistent across subgroups of age, sex, race and several comorbidities (all *P* for interaction > 0.05), demonstrating good population robustness. Adding logBAR to six traditional severity scoring systems (APACHE II, APS III, Charlson, OASIS, SAPS II and SOFA) significantly improved the predictive performance (AUC) of each score for 28-day mortality (DeLong test *P* < 0.01), suggesting that logBAR can serve as a useful supplement to existing scores.

From a mechanistic perspective, urosepsis is driven mainly by Gram-negative bacterial endotoxins that activate signaling pathways such as NF-κB, inducing systemic inflammatory responses and oxidative stress. This leads to renal tubular epithelial cell injury, microcirculatory dysfunction and tubuloglomerular feedback dysregulation, ultimately reducing glomerular filtration rate and causing BUN accumulation (33, 34). In addition, the hypercatabolic state makes BUN more sensitive to pre-renal factors and metabolic load (35). On the other hand, inflammatory cytokines (such as TNF-α, IL-1 and IL-6) inhibit hepatocyte albumin mRNA expression and reduce albumin synthesis (36). Systemic capillary leak syndrome promotes albumin extravasation, worsening hypoalbuminaemia (37). Thus, BUN and albumin contribute to disease progression through two intertwined pathological axes, kidney injury/metabolic load and inflammation/nutrition, making the combined assessment using BAR pathophysiologically reasonable. Importantly, BAR requires only two routine laboratory tests (serum BUN and albumin) that are readily available at admission, without the need for complex scoring tools. Therefore, logBAR is a prognostic marker, not a predictive or intervention-guiding biomarker; whether logBAR-guided interventions improve outcomes requires prospective validation. However, it should be noted that the magnitude of AUC improvement was modest (ΔAUC 0.021–0.079), and decision curve analysis showed that the additional net benefit of logBAR was limited, particularly in the external validation cohort where net benefit was negligible at most threshold probabilities. Therefore, logBAR should be regarded as a supplemental prognostic marker that may assist in risk stratification, rather than a standalone tool for clinical decision-making. The present study provides strong evidence for the clinical application of BAR in urosepsis through dual-cohort validation, a large sample size and incremental effect analysis over traditional scores.

Compared with previous studies (16–20, 23), this study has several notable strengths. First, most previous studies on the prognostic value of BAR focused on general sepsis populations, but the pathophysiological mechanisms differ substantially depending on the infection source, making it difficult to extrapolate those conclusions directly. The present study strictly focused on the specific subgroup of urosepsis and systematically validated the prognostic value of BAR, filling a gap in the field. Second, the only existing similar study in urosepsis had a sample size of only 156 patients, whereas the present study included 3,020 patients with urosepsis, approximately 20 times larger, greatly improving statistical power and robustness of the conclusions. Third, this study used a dual-cohort cross-validation design combining an internal discovery cohort from MIMIC-IV with a real-world external validation cohort. The association between logBAR and short-term mortality was confirmed in the internal cohort and supported in the external cohort, where the unadjusted association was significant (HR = 1.64, *P* = 0.024) and the dose–response relationship was confirmed (RCS *P* < 0.001). In the external cohort, the unadjusted association was significant (HR = 1.64, *P* = 0.024) and the adjusted association using a reduced model with adequate EPV (age, sex, lactate, creatinine; EPV = 10.3) also remained significant (HR = 2.23, *P* = 0.004), confirming the direction and magnitude of the association with statistical stability. Fourth, previous studies only reported the standalone predictive ability of BAR (AUC approximately 0.66–0.67) without evaluating its incremental value over existing clinical tools. For the first time, the present study added logBAR to six traditional severity scoring systems (APACHE II, APS III, Charlson, OASIS, SAPS II and SOFA). The results showed that adding logBAR significantly improved the predictive performance of each score (DeLong test *P* < 0.01), with AUC increases ranging from 0.024 to 0.079, highlighting its clinical value as an adjunctive assessment tool. Fifth, previous studies rarely provided a directly applicable clinical cut-off. The present study determined the optimal cut-off of logBAR for predicting 28-day ICU mortality in the derivation cohort using ROC curves and maximizing the Youden index, yielding a value of 2.344. This enhances the operability of rapid bedside risk stratification for high-risk patients.

However, several limitations of this study should be noted. First, the retrospective design cannot establish causality; despite multivariable adjustment, residual confounding may still exist, and prospective cohort studies are needed for validation. Second, BAR was based on a single measurement within 24 h of admission and did not capture dynamic changes in renal function and inflammatory-nutritional status during the ICU stay, particularly after disease fluctuations or therapeutic interventions (e.g., CRRT, vasoactive agents). Future studies should employ trajectory-based modeling approaches to explore the association between dynamic BAR trajectories and short-term prognosis. Third, the database lacked key information such as specific types of urological surgery, pathogen and susceptibility results, and mixed infections; these unmeasured variables could serve as potential confounders affecting the robustness of the conclusions. Fourth, the external validation was single-center with a relatively small sample size (only 41 death events), and clinical practices evolve over time, limiting the generalizability of the findings. Larger, multicenter prospective studies are required to further confirm the prognostic value of BAR. Fifth, although covariate selection was based on previous literature, selection bias may still exist; future validation studies should pre-specify covariates guided by directed acyclic graphs to enhance result reliability. Sixth, the optimal clinical cut-off for BAR has not been standardized. The present study used tertiles for subgroup analyses, but thresholds vary considerably across studies; a unified threshold should be established in the future to facilitate clinical adoption.

In sensitivity analyses, using stricter sepsis severity definitions (SOFA ≥ 6, lactate ≥2, and SIRS WBC criteria) in the internal cohort consistently confirmed the association between BAR and mortality risk, with HRs of 1.72 (95% CI: 1.46–2.02), 2.24 (95% CI: 1.85–2.71), and 1.95 (95% CI: 1.63–2.33), respectively, all *P* < 0.001. Sequential removal of potential pathway variables from Model 3 (including serum creatinine, AKI, CRRT, lactate, vasopressors, glucocorticoids, and analgesia/sedation) showed that the logBAR–mortality association remained robust across all adjustment sets, with HRs ranging from 1.77 to 1.98 and all VIFs < 2.2, ruling out overadjustment or collinearity. Regarding model performance, although adding logBAR significantly improved the discriminative ability of all severity scores (DeLong test *P* < 0.01), the magnitude of AUC improvement was modest (ΔAUC 0.021–0.079). Calibration analysis showed that internal cohort models were well calibrated (bootstrap optimism-corrected slopes 0.99–1.02); in the external cohort, adding logBAR tended to improve calibration slopes toward the ideal value of 1.0, although estimates were unstable due to the small number of external events (*n* = 41). Decision curve analysis demonstrated modest net benefit gains in the internal cohort at certain thresholds, whereas gains in the external cohort were negligible. Overall, logBAR provides incremental prognostic information, but its standalone clinical decision-making utility remains limited. These calibration and decision curve findings warrant further validation in larger, multicenter cohorts.

## Conclusion

This study, based on the large MIMIC-IV public database and a real-world cohort from the Affiliated Hospital of Guizhou Medical University, systematically evaluated the short-term prognostic value of BAR for the first time in a large sample of critically ill patients with urosepsis. The results demonstrated that logBAR was an independent risk factor for 28-day ICU mortality and in-hospital all-cause mortality, exhibiting a non-linear positive dose-response relationship with short-term mortality, and this association remained robust across subgroups of different demographic characteristics and comorbidities. Furthermore, adding logBAR to six traditional severity scores (APACHE II, APS III, Charlson, OASIS, SAPS II, and SOFA) significantly improved the predictive performance of each score. Meanwhile, this study identified the optimal cut-off value of logBAR for predicting 28-day ICU mortality as 2.344, providing a practical and actionable basis for rapid bedside risk stratification in clinical practice. Given that BAR is easy to calculate, low-cost, and provides immediate results, it holds promise as a useful supplementary tool for early risk stratification in patients with urosepsis. However, owing to its modest incremental predictive performance and the lack of clear net benefit observed in the external cohort, its independent clinical decision-making utility remains relatively limited. Therefore, further prospective multicenter studies are still warranted to validate the causal relationship of BAR and its practical value in dynamic monitoring.

## Data Availability

The original contributions presented in this study are included in the article/Supplementary material, further inquiries can be directed to the corresponding author.

## Supplementary material

Supplementary Figure 1 Subgroup and interaction analyses for the association between logBAR (as a continuous variable) and 28-day ICU mortality.

Supplementary Figure 2 Subgroup and interaction analyses for the association between logBAR (as a continuous variable) and 28-day hospital mortality.

Supplementary Figure 3 Calibration plots for all six severity scores (APACHE II, APS III, SAPS II, OASIS, Charlson, SOFA) for the prediction of 28-day ICU mortality, with and without the addition of logBAR, in the internal and external validation cohorts. Models were fitted separately within each cohort. Blue lines: score alone; orange lines: score plus logBAR; dashed diagonal: perfect calibration.

Supplementary Figure 4 Decision curve analysis for all six severity scores (APACHE II, APS III, SAPS II, OASIS, Charlson, SOFA) for the prediction of 28-day ICU mortality, with and without the addition of logBAR, in the internal and external validation cohorts. Models were fitted separately within each cohort. Blue lines: score alone; orange lines: score plus logBAR; dotted grey lines: treat-all; dashed horizontal line: treat-none.

Supplementary Table 1 Baseline characteristics of patients with urosepsis stratified by ICU survival status in the MIMIC-IV cohort. Data are presented as mean (SD), median [IQR], or n (%). *P*-values were calculated using Student’s *t*-test, Welch’s *t*-test, Mann-Whitney U test, or chi-square test as appropriate. AKI, acute kidney injury; CKD, chronic kidney disease; DM, diabetes mellitus; HLD, hyperlipidaemia; HF, heart failure; MI, myocardial infarction; IHD, ischaemic heart disease; COPD, chronic obstructive pulmonary disease; SA, analgesia/sedation; VP, vasopressor use; GC, glucocorticoid use; CRRT, continuous renal replacement therapy; SOFA, Sequential Organ Failure Assessment; APACHE II, Acute Physiology and Chronic Health Evaluation II; APS III, Acute Physiology Score III; SAPS II, Simplified Acute Physiology Score II; OASIS, Oxford Acute Severity of Illness Score; ALB, serum albumin; CREA, serum creatinine; UREA, blood urea; LDH, lactate dehydrogenase; Lac, lactate.

Supplementary Table 2 Baseline characteristics of patients with urosepsis stratified by ICU survival status in the external validation cohort (Affiliated Hospital of Guizhou Medical University).
