# Supplementary material for: Association of blood urea nitrogen to albumin ratio with short-term mortality in critically ill patients with urosepsis
Source: Front Med (Lausanne). 2026 Aug 18;13:1860492. doi: 10.3389/fmed.2026.1860492 (PMC13529655; doi:10.3389/fmed.2026.1860492)
Supplement: Supplementary file 1 [file Table_1.docx]

Summary descriptives table by groups of `ICU_dead'

|  | **ALL** | **Survivor** | **No-survivor** | **P value** |
| --- | --- | --- | --- | --- |
|  | ***N=3020*** | ***N=2576*** | ***N=444*** |  |
| Age | 70.5 (15.1) | 70.0 (15.2) | 73.2 (14.1) | <0.001 |
| Gender: | 1318 (43.6%) | 1121 (43.5%) | 197 (44.4%) | 0.777 |
| Race: | 1962 (65.0%) | 1690 (65.6%) | 272 (61.3%) | 0.086 |
| Weight | 82.1 (25.8) | 82.1 (25.7) | 82.3 (26.9) | 0.886 |
| HPY: | 1062 (35.2%) | 939 (36.5%) | 123 (27.7%) | <0.001 |
| AKI: | 1896 (62.8%) | 1538 (59.7%) | 358 (80.6%) | <0.001 |
| CKD: | 878 (29.1%) | 723 (28.1%) | 155 (34.9%) | 0.004 |
| DM: | 1141 (37.8%) | 978 (38.0%) | 163 (36.7%) | 0.652 |
| HLD: | 1059 (35.1%) | 932 (36.2%) | 127 (28.6%) | 0.002 |
| HF: | 1189 (39.4%) | 993 (38.5%) | 196 (44.1%) | 0.030 |
| MI: | 317 (10.5%) | 253 (9.82%) | 64 (14.4%) | 0.005 |
| IHD: | 1115 (36.9%) | 943 (36.6%) | 172 (38.7%) | 0.420 |
| COPD: | 495 (16.4%) | 407 (15.8%) | 88 (19.8%) | 0.041 |
| SOFA | 6.90 (3.48) | 6.60 (3.28) | 8.64 (4.05) | <0.001 |
| APSIII | 58.9 (21.0) | 56.8 (20.0) | 71.4 (22.4) | <0.001 |
| SAPSII | 45.0 (13.4) | 43.7 (12.8) | 52.8 (14.0) | <0.001 |
| OASIS | 36.0 (8.32) | 35.5 (8.11) | 38.7 (8.97) | <0.001 |
| Charlson | 6.31 (2.91) | 6.13 (2.86) | 7.35 (3.01) | <0.001 |
| APACHEII | 21.8 (7.00) | 21.2 (6.85) | 24.9 (7.06) | <0.001 |
| HR | 91.5 (21.3) | 91.4 (21.3) | 92.2 (21.1) | 0.433 |
| NBPS | 120 (25.8) | 121 (25.7) | 115 (25.8) | <0.001 |
| NBPD | 67.5 (19.8) | 67.9 (19.5) | 65.3 (21.0) | 0.018 |
| RR | 20.2 (6.41) | 20.1 (6.41) | 20.4 (6.43) | 0.400 |
| Spo2 | 96.5 (4.73) | 96.5 (4.68) | 96.0 (5.00) | 0.031 |
| HCT | 31.3 (6.63) | 31.5 (6.61) | 30.3 (6.67) | 0.001 |
| Hb | 10.1 (2.20) | 10.2 (2.20) | 9.80 (2.16) | 0.001 |
| PLT | 200 (118) | 202 (117) | 192 (123) | 0.110 |
| RDW | 16.1 (2.63) | 15.9 (2.52) | 17.0 (3.02) | <0.001 |
| RBC | 3.40 (0.78) | 3.42 (0.77) | 3.27 (0.80) | <0.001 |
| WBC | 13.9 (14.8) | 13.5 (14.0) | 16.0 (19.1) | 0.007 |
| ALB | 2.90 (0.62) | 2.93 (0.60) | 2.74 (0.67) | <0.001 |
| GA | 15.7 (4.73) | 15.6 (4.67) | 16.4 (5.03) | 0.001 |
| Ca | 8.27 (0.99) | 8.28 (0.99) | 8.24 (1.01) | 0.429 |
| Cl | 104 (7.94) | 104 (7.78) | 104 (8.79) | 0.375 |
| Glu | 154 (84.9) | 154 (85.0) | 151 (84.1) | 0.428 |
| K | 4.23 (0.80) | 4.20 (0.79) | 4.36 (0.84) | <0.001 |
| Na | 139 (6.77) | 139 (6.63) | 138 (7.55) | 0.599 |
| CO2CP | 23.9 (6.52) | 24.1 (6.52) | 22.7 (6.39) | <0.001 |
| LAC | 2.43 (2.04) | 2.36 (2.01) | 2.85 (2.15) | <0.001 |
| PCO2 | 42.3 (12.7) | 42.3 (12.7) | 42.0 (12.6) | 0.586 |
| PH | 7.35 (0.10) | 7.35 (0.10) | 7.33 (0.11) | <0.001 |
| PO2 | 117 (96.5) | 119 (97.7) | 106 (88.6) | 0.005 |
| INR | 1.40 [1.20;1.80] | 1.30 [1.20;1.70] | 1.50 [1.30;2.10] | <0.001 |
| PT | 15.1 [13.0;19.1] | 14.9 [12.9;18.6] | 16.6 [13.9;22.2] | <0.001 |
| PTT | 32.4 [27.9;41.4] | 32.1 [27.7;40.4] | 35.0 [28.8;47.5] | <0.001 |
| ALT | 25.0 [15.0;54.0] | 25.0 [15.0;53.0] | 28.0 [16.8;62.2] | 0.015 |
| AST | 40.0 [23.0;87.0] | 38.0 [23.0;82.0] | 50.5 [27.0;116] | <0.001 |
| TB | 0.70 [0.40;1.40] | 0.70 [0.40;1.40] | 0.90 [0.40;2.70] | <0.001 |
| CREA | 1.30 [0.80;2.20] | 1.30 [0.80;2.10] | 1.60 [1.00;2.62] | <0.001 |
| UREA | 29.0 [18.0;49.0] | 27.0 [17.0;46.0] | 40.0 [24.0;61.0] | <0.001 |
| LDH | 285 [213;415] | 279 [211;400] | 340 [241;549] | <0.001 |
| SA: | 2032 (67.3%) | 1687 (65.5%) | 345 (77.7%) | <0.001 |
| VP: | 1880 (62.3%) | 1530 (59.4%) | 350 (78.8%) | <0.001 |
| GC: | 980 (32.5%) | 807 (31.3%) | 173 (39.0%) | 0.002 |
| Ventilation: | 2610 (86.4%) | 2225 (86.4%) | 385 (86.7%) | 0.907 |
| CRRT: | 326 (10.8%) | 239 (9.28%) | 87 (19.6%) | <0.001 |
| CRRT: | 326 (10.8%) | 239 (9.28%) | 87 (19.6%) | <0.001 |
