# Supplementary material for: Association of blood urea nitrogen to albumin ratio with short-term mortality in critically ill patients with urosepsis
Source: Front Med (Lausanne). 2026 Aug 18;13:1860492. doi: 10.3389/fmed.2026.1860492 (PMC13529655; doi:10.3389/fmed.2026.1860492)
Supplement: Supplementary file 2 [file Table_2.docx]

Summary descriptives table by groups of `ICU_dead'

|  | **[ALL]** | **0** | **1** | **p.overall** |
| --- | --- | --- | --- | --- |
|  | ***N=415*** | ***N=374*** | ***N=41*** |  |
| logBAR | 2.35 (0.73) | 2.34 (0.75) | 2.50 (0.40) | 0.026 |
| Age | 70.6 (15.1) | 70.2 (15.3) | 74.3 (12.8) | 0.064 |
| Gender: |  |  |  | 0.546 |
| F | 236 (56.9%) | 215 (57.5%) | 21 (51.2%) |  |
| M | 179 (43.1%) | 159 (42.5%) | 20 (48.8%) |  |
| Weight | 68.2 (11.4) | 68.4 (11.5) | 66.4 (10.4) | 0.271 |
| Hpy: |  |  |  | 0.246 |
| No | 275 (66.3%) | 244 (65.2%) | 31 (75.6%) |  |
| Yes | 140 (33.7%) | 130 (34.8%) | 10 (24.4%) |  |
| AKI: |  |  |  | 0.078 |
| No | 159 (38.3%) | 149 (39.8%) | 10 (24.4%) |  |
| Yes | 256 (61.7%) | 225 (60.2%) | 31 (75.6%) |  |
| CKD: |  |  |  | 0.872 |
| No | 293 (70.6%) | 265 (70.9%) | 28 (68.3%) |  |
| Yes | 122 (29.4%) | 109 (29.1%) | 13 (31.7%) |  |
| DM: |  |  |  | 0.789 |
| No | 256 (61.7%) | 232 (62.0%) | 24 (58.5%) |  |
| Yes | 159 (38.3%) | 142 (38.0%) | 17 (41.5%) |  |
| HLD: |  |  |  | 0.381 |
| No | 273 (65.8%) | 243 (65.0%) | 30 (73.2%) |  |
| Yes | 142 (34.2%) | 131 (35.0%) | 11 (26.8%) |  |
| HF: |  |  |  | 0.376 |
| No | 312 (75.2%) | 284 (75.9%) | 28 (68.3%) |  |
| Yes | 103 (24.8%) | 90 (24.1%) | 13 (31.7%) |  |
| MI: |  |  |  | 1.000 |
| No | 399 (96.1%) | 359 (96.0%) | 40 (97.6%) |  |
| Yes | 16 (3.86%) | 15 (4.01%) | 1 (2.44%) |  |
| IHD: |  |  |  | 0.969 |
| No | 340 (81.9%) | 307 (82.1%) | 33 (80.5%) |  |
| Yes | 75 (18.1%) | 67 (17.9%) | 8 (19.5%) |  |
| COPD: |  |  |  | 0.373 |
| No | 349 (84.1%) | 317 (84.8%) | 32 (78.0%) |  |
| Yes | 66 (15.9%) | 57 (15.2%) | 9 (22.0%) |  |
| SOFA | 7.00 (3.54) | 6.87 (3.44) | 8.27 (4.25) | 0.047 |
| APSIII | 59.1 (20.8) | 58.4 (20.7) | 65.8 (21.1) | 0.037 |
| SAPSII | 45.6 (13.3) | 45.0 (13.3) | 51.1 (12.7) | 0.005 |
| OASIS | 36.0 (8.11) | 35.8 (8.04) | 37.6 (8.61) | 0.197 |
| Charlson | 6.32 (2.91) | 6.18 (2.83) | 7.59 (3.32) | 0.012 |
| APACHEII | 21.8 (7.30) | 21.6 (7.42) | 23.5 (5.95) | 0.063 |
| HR | 92.8 (20.3) | 92.9 (20.2) | 91.7 (21.4) | 0.723 |
| NBPS | 119 (25.7) | 119 (25.8) | 120 (24.9) | 0.727 |
| NBPD | 67.3 (19.7) | 67.1 (19.7) | 69.5 (19.2) | 0.462 |
| RR | 20.2 (6.38) | 20.2 (6.26) | 20.2 (7.47) | 0.995 |
| Spo2 | 96.5 (4.83) | 96.5 (4.92) | 96.6 (3.92) | 0.863 |
| HCT | 31.2 (6.71) | 31.3 (6.65) | 30.8 (7.35) | 0.697 |
| Hb | 10.1 (2.24) | 10.1 (2.24) | 9.90 (2.29) | 0.587 |
| PLT | 199 (113) | 199 (113) | 194 (115) | 0.807 |
| RDW | 16.2 (2.74) | 16.2 (2.72) | 16.9 (2.90) | 0.137 |
| RBC | 3.40 (0.78) | 3.40 (0.78) | 3.34 (0.84) | 0.644 |
| WBC | 13.8 (14.7) | 13.0 (7.38) | 20.9 (40.8) | 0.227 |
| ALB | 2.92 (0.63) | 2.92 (0.63) | 2.95 (0.64) | 0.757 |
| GA | 15.7 (4.58) | 15.8 (4.60) | 15.4 (4.38) | 0.647 |
| Ca | 8.29 (1.09) | 8.29 (1.10) | 8.28 (1.00) | 0.951 |
| Cl | 104 (7.73) | 104 (7.78) | 105 (7.22) | 0.324 |
| Glu | 159 (85.8) | 160 (89.0) | 153 (49.3) | 0.439 |
| K | 4.27 (0.85) | 4.27 (0.85) | 4.19 (0.85) | 0.528 |
| Na | 139 (6.37) | 139 (6.38) | 139 (6.28) | 0.484 |
| TCO2 | 24.3 (6.48) | 24.4 (6.61) | 23.3 (5.13) | 0.230 |
| Lac | 2.44 (2.02) | 2.40 (2.03) | 2.79 (1.89) | 0.221 |
| PCO2 | 43.1 (12.7) | 43.2 (12.8) | 42.5 (11.4) | 0.722 |
| PH | 7.35 (0.11) | 7.35 (0.11) | 7.34 (0.10) | 0.561 |
| PO2 | 124 (103) | 125 (103) | 115 (104) | 0.577 |
| INR | 1.70 (1.08) | 1.66 (1.06) | 2.02 (1.13) | 0.058 |
| PT | 18.3 (10.5) | 18.0 (10.3) | 21.7 (11.6) | 0.054 |
| PTT | 40.9 (25.0) | 40.2 (24.4) | 46.9 (29.7) | 0.170 |
| ALT | 149 (717) | 148 (740) | 153 (467) | 0.955 |
| AST | 213 (763) | 210 (783) | 236 (559) | 0.794 |
| TB | 2.05 (4.37) | 1.84 (3.89) | 4.01 (7.23) | 0.066 |
| CREA | 1.81 (1.49) | 1.84 (1.54) | 1.56 (0.91) | 0.094 |
| UREA | 37.1 (24.9) | 37.1 (25.9) | 37.1 (13.4) | 0.990 |
| LDH | 395 (399) | 389 (403) | 451 (362) | 0.305 |
| SA: |  |  |  | 0.619 |
| No | 141 (34.0%) | 129 (34.5%) | 12 (29.3%) |  |
| Yes | 274 (66.0%) | 245 (65.5%) | 29 (70.7%) |  |
| VP: |  |  |  | 0.250 |
| No | 161 (38.8%) | 149 (39.8%) | 12 (29.3%) |  |
| Yes | 254 (61.2%) | 225 (60.2%) | 29 (70.7%) |  |
| GC: |  |  |  | 0.292 |
| No | 288 (69.4%) | 263 (70.3%) | 25 (61.0%) |  |
| Yes | 127 (30.6%) | 111 (29.7%) | 16 (39.0%) |  |
| Ventilation: |  |  |  | 1.000 |
| No | 56 (13.5%) | 50 (13.4%) | 6 (14.6%) |  |
| Yes | 359 (86.5%) | 324 (86.6%) | 35 (85.4%) |  |
| CRRT: |  |  |  | 0.464 |
| No | 364 (87.7%) | 330 (88.2%) | 34 (82.9%) |  |
| Yes | 51 (12.3%) | 44 (11.8%) | 7 (17.1%) |  |
| ICU_day | 6.83 (7.21) | 6.85 (7.39) | 6.65 (5.43) | 0.832 |
